# Impact of next-generation hormonal agents on treatment patterns among patients with metastatic hormone-sensitive prostate cancer: a real-world study from the United States, five European countries and Japan

**DOI:** 10.1186/s12894-022-00979-9

**Published:** 2022-03-11

**Authors:** Andrea Leith, Amanda Ribbands, Jeri Kim, Emily Clayton, Liane Gillespie-Akar, Lingfeng Yang, Sameer R. Ghate

**Affiliations:** 1 Adelphi Real World, Bollington, UK; 2 Merck & Co., Inc., Kenilworth, NJ USA

**Keywords:** Metastatic hormone-sensitive prostate cancer, Metastatic castration-sensitive prostate cancer, Novel hormonal agents, Treatment patterns, Real-world evidence

## Abstract

**Background:**

Until five years ago, the metastatic hormone-sensitive prostate cancer (mHSPC) treatment landscape was dominated by the use of androgen deprivation therapy (ADT) alone. However, novel hormonal agents (NHAs) and chemotherapy are now approved for male patients with mHSPC. This study aimed to understand the impact NHA approvals had on mHSPC real-world treatment patterns and to identify the key factors associated with NHA or chemotherapy (± ADT) usage vs ADT alone.

**Methods:**

Data were collected from the Adelphi Prostate Cancer Disease Specific Programme (DSP)™, a point-in-time survey of physicians and their consulting patients conducted in the United States (US), five European countries (France, Germany, Italy, Spain, and the United Kingdom), and Japan between January and August 2020. Data were analysed using descriptive statistics for individual countries, regions, and all countries combined. Pairwise analyses were used to further investigate differences between treatment groups at global level.

**Results:**

336 physicians provided data on 1195 mHSPC patients. Globally, at data collection, the most common mHSPC regimen initiated first was ADT alone (47%), followed by NHAs (± ADT) (31%, of which 21% was abiraterone, 8% was enzalutamide, and 2% was apalutamide) and chemotherapy (± ADT) (19%). The highest rates of ADT alone usage were observed in Japan (78%) and Italy (66%), and the lowest in Spain (34%) and in the US (36%). Our results showed that clinical decision making was driven by patient fitness, compliance, tolerance of adverse events, and balance of impact on quality of life vs overall survival.

**Conclusions:**

This real-world survey offered early insights into the evolving mHSPC treatment paradigm. It showed that in 2020, ADT alone remained the most common initial mHSPC therapy, suggesting that physicians may prefer using treatments which they are familiar and have experience with, despite clinical trial evidence of improved survival with NHAs or chemotherapy (± ADT) vs ADT alone. Results also indicated that physicians prescribed specific mHSPC treatments primarily based on the following criteria: patient preference, disease burden/severity, and the performance status and comorbidities of the patient. To fully appreciate the rapidly changing mHSPC treatment landscape and monitor NHA uptake, additional real-world studies are required.

## Background

Prostate cancer remains one of the leading causes of death among men worldwide [1]. Up to one-third of patients develop metastatic prostate cancer at some point in the course of their disease [2], with metastatic castration-resistant prostate cancer (mCRPC) being associated with poor prognosis and high mortality [3].

One of the disease states that precedes mCRPC is known as metastatic hormone-sensitive prostate cancer (mHSPC) or metastatic castration-sensitive/hormone-naïve disease and encompasses a heterogenous patient population with varying levels of disease biology, burden of disease, functional status, cancer-related symptoms, and outcomes [2]. De novo metastases are not uncommon; for example, they have been reported in 63% of patients in community oncology settings in the United States (US) [4]. This may be because prostate cancer screening is not routine and hence many patients end up presenting with later stage, locally advanced or metastatic disease [5, 6]. Patients with de novo mHSPC may have more aggressive disease and poorer outcomes than patients who develop metastases later in the course of the disease [7].

After years of little advancement, it is only in the past five years that the treatment landscape for mHSPC has experienced important developments [2, 3, 8, 9]. For seven decades, androgen deprivation therapy (ADT) alone, which was first introduced in the 1940s, was standard of care (SOC) for patients with mHSPC. It was not until 2015 that the therapeutic armamentarium began to develop, when docetaxel chemotherapy (CHAARTED [10]) demonstrated a significant overall survival (OS) benefit compared with ADT alone in mHSPC. Since then, the mHSPC treatment space has been rapidly evolving, with the introduction of novel hormonal agents (NHAs) abiraterone (STAMPEDE [11]; LATITUDE [12]), enzalutamide (ENZAMET [13]), and apalutamide (TITAN [14]). While the former two were originally approved and indicated for use in patients with mCRPC, all of them demonstrated superior OS benefit when added to ADT vs ADT alone or in combination with placebo or nonsteroidal antiandrogen [2, 3, 8].

Abiraterone, apalutamide and enzalutamide are now approved in the US, the European Union (EU), and Japan [15–24]. In addition, docetaxel chemotherapy is approved in the EU [25]. Specific approval dates can be found in Table 1.

**Table 1:** Docetaxel and NHA approval dates and indications in mHSPC

Drug name	Country	Approval date	Indication
Docetaxel	US	N/A	N/A
	EU (EMA) [25]	Sept 2019	In combination with ADT, with or without prednisone or prednisolone, are indicated for the treatment of patients with mHSPC
	Japan	N/A	N/A
Abiraterone	US (FDA) [17, 22]	Feb 2018	In combination with prednisone for the treatment of high-risk patients with mHSPC
	EU (EMA) [15]	Nov 2017	In combination with prednisone or prednisolone for the treatment of newly diagnosed high risk mHSPC in adult men in combination with androgen deprivation therapy (ADT)
	Japan (MHLW) [23]	Feb 2018	For the treatment of hormone-naïve prostate cancer (HNPC) with high risk prognostic factors
Apalutamide	US (FDA) [19]	Sept 2019	For the treatment of patients with mHSPC
	EU (EMA) [16]	Jan 2020	Adult men with mHSPC in combination with ADT
	Japan (MHLW) [24]	May 2020	For the treatment of men with prostate cancer with distant metastases
Enzalutamide	US (FDA) [18]	Dec 2019	For the treatment of patients with mHSPC
	EU (EMA) [21]	May 2021	For the treatment of patients with mHSPC
	Japan (MHLW) [20]	May 2020	For the treatment of prostate cancer patients with distant metastasis

*US* United States, *EU *Europe, *EMA* European Medicines Agency, *FDA* Food and Drug Administration, *MHLW* Japan Ministry of Health, Labour and Welfare

All agents are now included in updated guidelines from the European Association of Urology [26], European Society for Oncology [27], National Comprehensive Cancer Network® (NCCN®) [28], and American Urological Association/American Society for Radiation Oncology/Society of Urologic Oncology [29]. As the mHSPC treatment landscape continues to evolve, the optimal treatment strategy is likely to vary by disease burden, patient demographics and clinical characteristics, as well as cost, the latter being particularly relevant in low- and middle-income countries [9]. As NHAs have been approved only relatively recently, there are currently few real-world published data assessing NHA use in the mHSPC space [30]; these are restricted to the US, and largely based on data collected prior to NHA approvals for mHSPC. They indicate that, despite the emergence of docetaxel or NHA combination therapies, a large proportion of men continue to be treated with ADT alone [30–33].

To the best of our knowledge, the present survey represents the first real-world investigation to evaluate mHSPC treatment patterns in different regions of the world post NHA approval. Its main objectives were to understand the impact of the approval of NHAs in the mHSPC space by describing real-world treatment patterns (initial regimens), patient demographics, and clinical characteristics among patients with mHSPC in the US, five European countries (EU5: France, Germany, Italy, Spain, and the United Kingdom [UK]), and Japan, and to identify the key factors associated with NHA or chemotherapy (± ADT) usage vs ADT alone.

## Methods

### Study design

Data were drawn from the Adelphi Prostate Cancer Disease Specific Programme (DSP)™, conducted in the US, EU5, and Japan between January and August 2020. DSPs are large, point-in-time surveys of physicians and their patients presenting in a real-world clinical setting, whose methodology has been previously published and validated [34–36].

### Participants

Physicians were identified by local fieldwork agents using physician panels and publicly available lists, and invited to participate if they had a specialty in medical oncology or urology, or were specialist surgeons; had personal responsibility for prescribing decisions; were seeing four or more patients (two or more patients in Japan) with metastatic prostate cancer per month, two of whom had to be diagnosed with mHSPC (one patient in Japan); and agreed to adhere to all survey rules and regulations.

### Data collection

Participating physicians completed detailed electronic patient record forms (PRFs) for the next four (three in Japan) consecutive consulting patients with mHSPC, reflective of real-world clinical practice. The PRFs collected detailed information about patient demographics, clinical characteristics, and patient management, including treatment history at the time of data collection. A list of definitions and derived variables used in the interpretation of these study data is given in Table 2.

**Table 2:** List of definitions and derived variables used in the study

Initial mHSPC treatment	The first treatment that a patient received in the mHSPC setting
NHA drugs	abiraterone, enzalutamide, apalutamide, darolutamide
Chemotherapy drugs	cabazitaxel, docetaxel, mitoxantrone, cisplatin, paclitaxel, carboplatin, etoposide, estramustine, ifosfamide
ADT drugs	degarelix, goserelin, leuprorelin, triptorelin, histrelin, bicalutamide, flutamide, nilutamide, ketoconazole, chlormadinone, cyproterone, buserelin
Immunotherapy drugs	sipuleucel-T, pembrolizumab
Radiotherapy drugs	radium-223, strontium 89
Corticosteroid drugs	prednisone, prednisolone, methylprednisolone, hydrocortisone, dexamethasone, betamethasone
Other drugs	diethylstilbestrol, padeliporfin, peplomycin sulfate, any other drug therapies not previously specified
NHA (± ADT)	Patients who received NHA (± ADT)
Chemotherapy (± ADT)	Patients who received chemotherapy (± ADT)
ADT alone	Patients who received ADT alone
Other combination including NHA	Patients who received any NHA drugs (not as a NHA (± ADT))
Chemotherapy combination	Patient who received a chemotherapy drug (not as a chemotherapy (± ADT)) in their initial mHSPC treatment. This does not include patients who received an NHA (± ADT) or NHA in combination
Any other combination	Patients who received a treatment not captured in the NHA (± ADT), chemotherapy (± ADT), ADT alone, other combination including NHA or chemotherapy combination treatment groups
Any NHA	Patients who received a NHA drug as either (± ADT) or in combination (± ADT)
No NHA	Patients who did not receive a NHA drug as either (± ADT) or in combination (± ADT)
High-risk disease	Patients with two of the following: Gleason score of 8 + , presence of visceral metastases, or 3 + bone lesions
Low-risk disease	Patients with ≤ 1 of the following: Gleason score of 8 + , presence of visceral metastases, or 3 + bone lesions
High disease volume	Patients with either of the following: presence of visceral metastases, or 4 + bone lesions with ≥ 1 beyond the vertebral bodies/pelvis
Low disease volume	Patients with neither of the following: presence of visceral metastases, nor 4 + bone lesions with ≥ 1 beyond the vertebral bodies/pelvis

*ADT* androgen deprivation therapy, *mHSPC* metastatic hormone-sensitive prostate cancer, *NHA* novel hormonal agent

To be included, patients had to meet the following eligibility criteria at the time of data collection: being mHSPC diagnosed aged 18 years or older; receiving systemic drug treatment for their mHSPC; having never participated in a clinical trial; receiving any line of therapy for mHSPC treatment (i.e., initial or subsequent treatment).

### Variables

The following variables of interest were collected or derived: patient demographics and clinical characteristics (age, employment status, Eastern Cooperative Oncology Group [ECOG] performance status, disease status, risk status, disease volume, sites of metastases, mean number of bone metastases, family history of prostate cancer, prostate-specific antigen [PSA], haemoglobin and alkaline phosphatase levels); initial treatment regimens for mHSPC; physician-reported drivers of initial mHSPC treatment choice (key clinical reasons for treatment choice).

### Analysis

Descriptive statistics for demographics, clinical characteristics, and treatment patterns data were reported at individual country level, as well as at regional level (i.e., US, aggregated EU5 data, and Japan), and for all countries combined (i.e., aggregated global data). Chi-squared and ANOVA tests were used to test across all groups.

To further investigate differences between treatment groups following descriptive analysis of treatment patterns, pairwise analyses (t-tests or Chi-squared tests) were conducted: NHA (± ADT) vs chemotherapy (± ADT), NHA (± ADT) vs ADT alone, and chemotherapy (± ADT) vs ADT alone. No adjustments for multiplicity were made. For clinical reasons, we specifically focused on the NHA (± ADT) vs ADT alone, and chemotherapy (± ADT) vs ADT alone treatment groups; these data are presented in detail in the text and in tables and figures. In addition, NHA (± ADT) vs chemotherapy (± ADT) results can also be found in Table 4. For the pairwise analyses, this article presented aggregated global data and findings were interpreted by treatment type at global level. Analyses were performed on a complete case basis using Stata 16.1 [37].

## Results

Globally, 336 physicians participated in the survey: 226 in the EU5 (France: 51; Germany: 50; Italy: 45; Spain: 45; UK: 35), 58 in the US, and 52 in Japan. Almost three-quarters (70.8%; n = 238) of physicians at global level were medical/clinical/radio oncologists, while 28.3% (n = 95) were urologists, and 0.9% (n = 3) were prostate/specialist cancer surgeons. The same pattern (i.e., oncologist as the majority specialty) was observed at EU5 and individual country level, except for Japan, where almost all physicians were urologists (92.3%; n = 48). At a global level, similar percentages of physicians worked primarily in an academic/cancer centre (49.7%) or a community setting (50.3%), and a similar pattern was observed in the US, Japan, and France. More physicians in Spain and the UK were based in academic/cancer centers compared with community settings (Spain: 84.4% vs 15.6%; UK: 80.0% vs 20.0%). The opposite was observed in Germany and Italy, where more physicians were community based (Germany: 80.0% vs 20.0%; Italy: 62.2% vs 37.8%) (data not shown).

Physicians completed PRFs for a total of 1195 patients: 182 in the US, 888 in the EU5 (France: 254; Germany: 179; Spain: 173; Italy: 155; UK: 127), and 125 in Japan.

### Patient demographics and clinical characteristics

Patient demographics and clinical characteristics are presented in Table 3. Pairwise analyses revealed several significant differences between treatment groups (NHA [± ADT] vs chemotherapy [± ADT], NHA [± ADT] vs ADT alone, and chemotherapy [± ADT] vs ADT alone), including for most recent PSA level, risk status and disease volume (Table 4). Men treated with NHA (± ADT) or chemotherapy (± ADT) vs ADT alone had significantly higher PSA levels (mean ± standard deviation [SD], 25.4 ± 77.9 ng/mL; 27.4 ± 74.0 ng/mL, respectively vs 13.9 ± 37.4 ng/mL, *p* ≤ 0.006). Similarly, significantly higher proportions of patients receiving NHAs or chemotherapy (± ADT) vs ADT alone had high-risk status (42.3%, n = 145; 63.1%, n = 135, respectively vs 27.7%, n = 144; both *p* < 0.001) and high disease volume (43.1%, n = 138; 65.5%, n = 133, respectively vs 28.2%, n = 129; both *p* < 0.001).

**Table 3:** Patient demographics and clinical characteristics

	Global (n = 1195)	UK (n = 127)	France (n = 254)	Germany (n = 179)	Italy (n = 155)	Spain (n = 173)	EU5 (n = 888)	Japan (n = 125)	US (n = 182)
Age, mean, years (SD)	72.1 (8.01)	70.7 (7.75)	73.7 (7.97)	70.3 (6.82)	73.7 (8.10)	71.3 (8.08)	72.1 (7.89)	75.3 (7.46)	69.4 (8.10)
Employment status at time of data collection, n (%)									
Working	158 (13.2)	24 (18.9)	5 (2.0)	11 (6.1)	17 (11.0)	12 (6.9)	69 (7.8)	27 (21.6)	62 (34.1)
Not working	1015 (84.9)	102 (80.3)	247 (97.2)	168 (93.9)	132 (85.2)	157 (90.8)	806 (90.8)	90 (72)	119 (65.4)
Don’t know	22 (1.8)	1 (0.8)	2 (0.8)	0 (0.0)	6 (3.9)	4 (2.3)	13 (1.5)	8 (6.4)	1 (0.5)
ECOG performance status at time of data collection, n (%)									
0–1	941 (78.7)	111 (87.4)	186 (73.2)	118 (65.9)	125 (80.6)	152 (87.9)	692 (77.9)	114 (91.2)	135 (74.2)
2–4	248 (20.8)	16 (12.6)	64 (25.2)	61 (34.1)	28 (18.1)	21 (12.1)	190 (21.4)	11 (8.8)	47 (25.8)
Unknown/not assessed	6 (0.5)	0 (0.0)	4 (1.6)	0 (0.0)	2 (1.3)	0 (0.0)	6 (0.7)	0 (0.0)	0 (0.0)
Disease status at time of data collection, n (%)	1189	126	252	178	153	173	882	125	182
Disease progressing	55 (4.6)	1 (0.8)	2 (0.8)	29 (16.3)	3 (2.0)	7 (4.0)	42 (4.8)	3 (2.4)	10 (5.5)
Stable	444 (37.3)	19 (15.1)	76 (30.2)	76 (42.7)	66 (43.1)	53 (30.6)	290 (32.9)	75 (60)	79 (43.4)
Responding to treatment	690 (58.0)	106 (84.1)	174 (69.0)	73 (41.0)	84 (54.9)	113 (65.3)	550 (62.4)	47 (37.6)	93 (51.1)
Patient’s family history of prostate cancer, n (%)	1195	127	254	179	155	173	888	125	182
Has a family history	111 (9.3)	9 (7.1)	18 (7.1)	30 (16.8)	8 (5.2)	17 (9.8)	82 (9.2)	5 (4.0)	24 (13.2)
Does not have a family history	997 (83.4)	106 (83.5)	224 (88.2)	142 (79.3)	130 (83.9)	150 (86.7)	752 (84.7)	99 (79.2)	146 (80.2)
Don’t know	87 (7.3)	12 (9.4)	12 (4.7)	7 (3.9)	17 (11.0)	6 (3.5)	54 (6.1)	21 (16.8)	12 (6.6)
Sites of metastases at time of data collection, n (%)									
Bone	1020 (85.4)	118 (92.9)	239 (94.1)	136 (76)	135 (87.1)	160 (92.5)	788 (88.7)	109 (87.2)	123 (67.6)
Brain	8 (0.7)	1 (0.8)	1 (0.4)	3 (1.7)	0 (0.0)	0 (0.0)	5 (0.6)	1 (0.8)	2 (1.1)
Lung	97 (8.1)	12 (9.4)	25 (9.8)	11 (6.1)	10 (6.5)	17 (9.8)	75 (8.4)	5 (4.0)	17 (9.3)
Pancreas	4 (0.3)	0 (0.0)	1 (0.4)	0 (0.0)	0 (0.0)	0 (0.0)	1 (0.1)	0 (0.0)	3 (1.6)
Liver	73 (6.1)	7 (5.5)	25 (9.8)	11 (6.1)	6 (3.9)	11 (6.4)	60 (6.8)	1 (0.8)	12 (6.6)
Adrenal glands	17 (1.4)	0 (0.0)	5 (2.0)	4 (2.2)	2 (1.3)	3 (1.7)	14 (1.6)	1 (0.8)	2 (1.1)
Peritoneal	30 (2.5)	0 (0.0)	5 (2.0)	10 (5.6)	2 (1.3)	0 (0.0)	17 (1.9)	0 (0.0)	13 (7.1)
Non-regional/distant lymph nodes	368 (30.8)	38 (29.9)	86 (33.9)	57 (31.8)	55 (35.5)	63 (36.4)	299 (33.7)	23 (18.4)	46 (25.3)
Other	6 (0.5)	0 (0.0)	1 (0.4)	1 (0.6)	0 (0.0)	1 (0.6)	3 (0.3)	0 (0.0)	3 (1.6)
Number of bone metastases sites identified, n (%)	850	76	209	117	111	139	652	94	104
1–3 lesions	435 (51.2)	36 (47.4)	104 (49.8)	60 (51.3)	54 (48.6)	60 (43.2)	314 (48.2)	57 (60.6)	64 (61.5)
4 + lesions	415 (48.8)	40 (52.6)	105 (50.2)	57 (48.7)	57 (51.4)	79 (56.8)	338 (51.8)	37 (39.4)	40 (38.5)
Risk status, n (%)									
Low-risk	674 (56.4)	58 (45.7)	133 (52.4)	115 (64.2)	94 (60.6)	89 (51.4)	489 (55.1)	55 (44.0)	130 (71.4)
High-risk	433 (36.2)	49 (38.6)	103 (40.6)	54 (30.2)	44 (28.4)	78 (45.1)	328 (36.9)	60 (48.0)	45 (24.7)
Don’t know	88 (7.4)	20 (15.7)	18 (7.1)	10 (5.6)	17 (11.0)	6 (3.5)	71 (8.0)	10 (8.0)	7 (3.8)
Disease volume, n (%)									
Low volume	598 (50.0)	50 (39.4)	117 (46.1)	102 (57.0)	67 (43.2)	76 (43.9)	412 (46.4)	77 (61.6)	109 (59.9)
High volume	409 (34.2)	34 (26.8)	104 (40.9)	54 (30.2)	58 (37.4)	75 (43.4)	325 (36.6)	33 (26.4)	51 (28.0)
Don’t know	188 (15.7)	43 (33.9)	33 (13.0)	23 (12.8)	30 (19.4)	22 (12.7)	151 (17.0)	15 (12.0)	22 (12.1)
Most recent PSA result at time of data collection (ng/mL), n	1037	101	215	158	139	161	774	116	147
Mean (SD)	20.1 (60.42)	30.8 (81.17)	26.5 (70.03)	20.8 (67.75)	11.3 (17.84)	18.4 (38.90)	21.5 (59.59)	7.4 (20.95)	22.7 (81.32)
Most recent haemoglobin test result at time of data collection (g/dL), n	615	66	116	74	87	133	476	79	60
Mean (SD)	12.0 (1.48)	11.7 (1.44)	12.3 (1.29)	11.8 (1.71)	11.8 (1.29)	12.0 (1.44)	12.0 (1.44)	12.3 (1.36)	12.3 (1.87)
Most recent alkaline phosphatase result at time of data collection (U/L), n	539	65	99	42	71	115	392	93	54
Mean (SD)	196.8 (141.55)	179.2 (118.50)	181.5 (132.55)	162.0 (94.58)	209.2 (187.94)	189.2 (134.52)	186.3 (139.34)	256.8 (146.57)	169.3 (115.83)

*ECOG* Eastern Cooperative Oncology Group, *EU5* France, Germany, Italy, Spain, and the United Kingdom, *PSA* prostate-specific antigen, *SD* standard deviation, *UK* United Kingdom, *US* United States

**Table 4:** Patient demographics and clinical characteristics: treatment group differences

	NHA (± ADT) (n = 367)	Chemo (± ADT) (n = 231)	ADT alone (n = 564)	*p*-value	Test type	Pairwise *p*-values
Age, mean, years (SD)	71.4 (7.1)	67.4 (7.2)	74.7 (7.7)	< 0.0001	AN	NHA vs Chemo: *p* < 0.001 NHA vs ADT alone: *p* < 0.001 Chemo vs ADT alone: *p* < 0.001
Employment status at time of data collection, n (%)	363	226	551	< 0.0001	CH	NHA vs Chemo: *p* = 0.182 NHA vs ADT alone: *p* = 0.008 Chemo vs ADT alone: *p* < 0.001
Working full-time	28 (7.7)	18 (8.0)	34 (6.2)			
Working part-time	25 (6.9)	17 (7.5)	24 (4.4)			
On long term sick leave	18 (5.0)	23 (10.2)	8 (1.5)			
Homemaker	2 (0.6)	0 (0.0)	8 (1.5)			
Student	1 (0.3)	0 (0.0)	0 (0.0)			
Retired	279 (76.9)	159 (70.4)	459 (83.3)			
Unemployed	10 (2.8)	9 (4.0)	18 (3.3)			
ECOG performance status at time of data collection, n (%)	367	230	559	0.0047	CH	NHA vs Chemo: *p* = 0.309 NHA vs ADT alone: *p* < 0.001 Chemo vs ADT alone: *p* = 0.208
0	80 (21.8)	65 (28.3)	185 (33.1)			
1	217 (59.1)	121 (52.6)	252 (45.1)			
2	58 (15.8)	38 (16.5)	96 (17.2)			
3	10 (2.7)	6 (2.6)	21 (3.8)			
4	2 (0.5)	0 (0.0)	5 (0.9)			
Disease status at time of data collection, n (%)	367	231	558	0.0129	CH	NHA vs Chemo: *p* = 0.170 NHA vs ADT alone: *p* = 0.060 Chemo vs ADT alone: *p* = 0.010
Disease progressing	21 (5.7)	10 (4.3)	17 (3.0)			
Stable	131 (35.7)	68 (29.4)	228 (40.9)			
Responding to treatment	215 (58.6)	153 (66.2)	313 (56.1)			
Patient’s family history of prostate cancer, n (%)	351	215	510	0.3223	CH	NHA vs Chemo: *p* = 0.376 NHA vs ADT alone: *p* = 0.140 Chemo vs ADT alone: *p* = 0.770
Has a family history	41 (11.7)	20 (9.3)	44 (8.6)			
Does not have a family history	310 (88.3)	195 (90.7)	466 (91.4)			
Most recent PSA result at time of data collection (ng/mL), n	325	201	485	0.0061	AN	NHA vs Chemo: *p* = 0.773 NHA vs ADT alone: *p* = 0.005 Chemo vs ADT alone: *p* = 0.002
Mean (SD)	25.4 (77.9)	27.4 (74.0)	13.9 (37.4)			
Most recent haemoglobin test result at time of data collection(g/dL), n	186	139	285	0.0148	AN	NHA vs Chemo: *p* = 0.006 NHA vs ADT alone: *p* = 0.045 Chemo vs ADT alone: *p* = 0.205
Mean (SD)	12.3 (1.5)	11.8 (1.4)	12 (1.4)			
Most recent alkaline phosphatase result at time of data collection (U/L), n	153	131	251	0.1779	AN	NHA vs Chemo: *p* = 0.160 NHA vs ADT alone: *p* = 0.868 Chemo vs ADT alone: *p* = 0.065
Mean (SD)	202.4 (152.3)	177.5 (144)	204.8 (133.3)			
Risk status, n (%)	343	214	520	< 0.0001	CH	NHA vs Chemo: *p* < 0.001 NHA vs ADT alone: *p* < 0.001 Chemo vs ADT alone: *p* < 0.001
Low-risk	198 (57.7)	79 (36.9)	376 (72.3)			
High-risk	145 (42.3)	135 (63.1)	144 (27.7)			
Disease volume, n (%)	320	203	457	< 0.0001	CH	NHA vs Chemo: *p* < 0.001 NHA vs ADT alone: *p* < 0.001 Chemo vs ADT alone: *p* < 0.001
Low volume	182 (56.9)	70 (34.5)	328 (71.8)			
High volume	138 (43.1)	133 (65.5)	129 (28.2)			
Presence of bone metastases, n (%)	367	231	564	0.1442	CH	NHA vs Chemo: *p* = 0.049 NHA vs ADT alone: *p* < 0.349 Chemo vs ADT alone: *p* < 0.183
Patient has bone metastases	306 (83.4)	206 (89.2)	483 (85.6)			
Patient does not have bone metastases	61 (16.6)	25 (10.8)	81 (14.4)			
Presence of brain metastases, n (%)				0.2747	CH	NHA vs Chemo: *p* = 0.134 NHA vs ADT alone: *p* = 0.554 Chemo vs ADT alone: *p* = 0.257
Patient has brain metastases	1 (0.3)	3 (1.3)	3 (0.5)			
Patient does not have brain metastases	366 (99.7)	228 (98.7)	561 (99.5)			
Presence of lung metastases, n (%)				< 0.0001	CH	NHA vs Chemo: *p* = 0.001 NHA vs ADT alone: *p* = 0.002 Chemo vs ADT alone: *p* < 0.001
Patient has lung metastases	32 (8.7)	41 (17.7)	22 (3.9)			
Patient does not have lung metastases	335 (91.3)	190 (82.3)	542 (96.1)			
Presence of pancreatic metastases, n (%)				0.1105	CH	NHA vs Chemo: *p* = 0.074 NHA vs ADT alone: *p* = 0.420 Chemo vs ADT alone: *p* = 0.151
Patient has pancreatic metastases	0 (0.0)	2 (0.9)	1 (0.2)			
Patient does not have pancreatic metastases	367 (100.0)	229 (99.1)	563 (99.8)			
Presence of liver metastases, n (%)				< 0.0001	CH	NHA vs Chemo: *p* = 0.002 NHA vs ADT alone: *p* < 0.001 Chemo vs ADT alone: *p* < 0.001
Patient has liver metastases	26 (7.1)	35 (15.2)	11 (2.0)			
Patient does not have liver metastases	341 (92.9)	196 (84.8)	553 (98.0)			
Presence of adrenal gland metastases, n (%)				0.0026	CH	NHA vs Chemo: *p* = 0.670 NHA vs ADT alone: *p* < 0.001 Chemo vs ADT alone: *p* = 0.003
Patient has adrenal gland metastases	10 (2.7)	5 (2.2)	1 (0.2)			
Patient does not have adrenal gland metastases	357 (97.3)	226 (97.8)	563 (99.8)			
Presence of peritoneal metastases, n (%)				0.0001	CH	NHA vs Chemo: *p* = 0.667 NHA vs ADT alone: *p* < 0.001 Chemo vs ADT alone: *p* < 0.001
Patient has peritoneal metastases	17 (4.6)	9 (3.9)	3 (0.5)			
Patient does not have peritoneal metastases	350 (95.4)	222 (96.1)	561 (99.5)			
Presence of non-regional/distant lymph node metastases, n (%)				0.0019	CH	NHA vs Chemo: *p* = 0.683 NHA vs ADT alone: *p* = 0.004 Chemo vs ADT alone: *p* = 0.003
Patient has non-regional/distant lymph node metastases	129 (35.1)	85 (36.8)	148 (26.2)			
Patient does not have non-regional/distant lymph node metastases	238 (64.9)	146 (63.2)	416 (73.8)			
Presence of other metastases, n (%)				0.6136	CH	NHA vs Chemo: *p* = 0.317 NHA vs ADT alone: *p* = 0.554 Chemo vs ADT alone: *p* = 0.589
Patient has other metastases	366 (99.7)	229 (99.1)	561 (99.5)			
Patient does not have other metastases	1 (0.3)	2 (0.9)	3 (0.5)			

*ADT* androgen deprivation therapy, *AN* ANOVA, *CH* Chi-squared, *Chemo* chemotherapy, *ECOG* Eastern Cooperative Oncology Group, *NHA* novel hormonal agents, *PSA* prostate-specific antigen, *SD* standard deviation

### Most common initial mHSPC treatments received at time of data collection

**Fig. 1:**
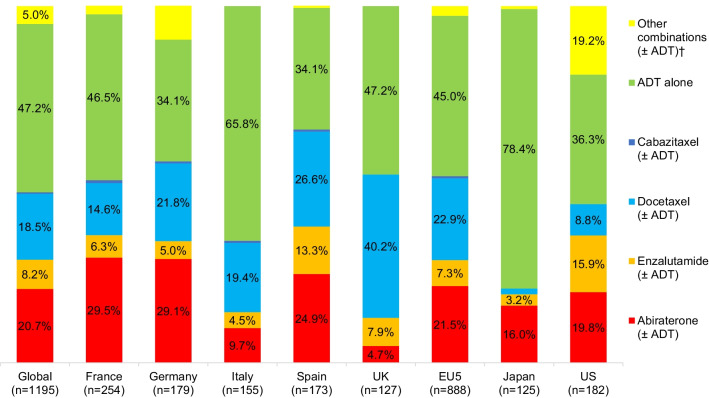
Initial mHSPC treatment received at time of data collection, split by regions. *Note*: Individual data labels that were < 3% are not shown. ADT: androgen deprivation therapy; EU5: France, Germany, Italy, Spain, and the United Kingdom; mHSPC: metastatic hormone-sensitive prostate cancer; NHA: novel hormonal agents (abiraterone, enzalutamide, apalutamide, darolutamide); UK: United Kingdom; US: United States. ^†^Other combinations included ‘Other NHAs’ that were being used in 1.7% of patients overall across treatment lines; ‘Other chemotherapy’ that was being used in 0.4% of patients overall across treatment lines; ‘Other combinations including NHA’ that were being used in 1.5% of patients overall across treatment lines; ‘Chemotherapy combination’ which was being used in 0.3% of patients overall across treatment lines; ‘Any other treatment combinations’ that were being used in 1% of patients overall across treatment lines

Globally (n = 1195), at the time of data collection, the most common mHSPC regimen initiated first was ADT alone (47.2%; n = 564), followed by NHA (± ADT) (30.7%; n = 367, of which 20.7% [n = 247] was abiraterone, 8.2%; [n = 98] was enzalutamide and 1.7% [n = 20] was apalutamide), and chemotherapy (± ADT) (19.3%; n = 231, mainly docetaxel [18.5%; n = 221]). Similar patterns were observed in the EU5 and Japan, but these were slightly different in the US (Fig. 1).

In the EU5 (n = 888), the most frequent mHSPC regimen initiated first was ADT alone (45.0%; n = 400), followed by NHA (± ADT) (30.1%; n = 267, of which 21.5%; n = 191 was abiraterone, 7.3%; [n = 65] was enzalutamide and 1.2% [n = 11] was apalutamide) and chemotherapy (± ADT) (23.4%; n = 208). NHA (± ADT) use differed across the EU5: Spain (38.2%; n = 66), France (37.8%; n = 96) and Germany (37.4%; n = 67) had the highest use, whereas Italy (14.2%; n = 22) and the UK (12.6%; n = 16) had the lowest use. Chemotherapy (± ADT) use also differed across the EU5, with the UK having the highest (40.2%; n = 51) and France the lowest (15.4%; n = 39) rate; across all EU5 countries, docetaxel was the most common chemotherapy drug.

In Japan, most mHSPC patients initiated ADT alone (78.4%; n = 98), followed by NHA (± ADT) (20.0%; n = 25, of which 16.0% [n = 20] was abiraterone, 3.2% [n = 4] was enzalutamide and 0.8% [n = 1] was apalutamide). Chemotherapy use was low (1.6%; n = 2).

By contrast, in the US the most common mHSPC regimen initiated was NHA (± ADT) (41.2%; n = 75], of which 19.8% (n = 36] was abiraterone, 15.9% [n = 29] was enzalutamide, 4.4% [n = 8] was apalutamide and 1.1% [n = 2] was darolutamide), followed by ADT alone (36.3%; n = 66) and chemotherapy (± ADT) (11.5%; n = 21).

### *Key factors associated with NHA use: NHA (*± *ADT) vs ADT alone*

Patients receiving NHA (± ADT) were significantly younger (71.4 ± 7.1 years) than patients receiving ADT alone (74.7 ± 7.7 years; *p* < 0.001) (Table 4).

**Fig. 2:**
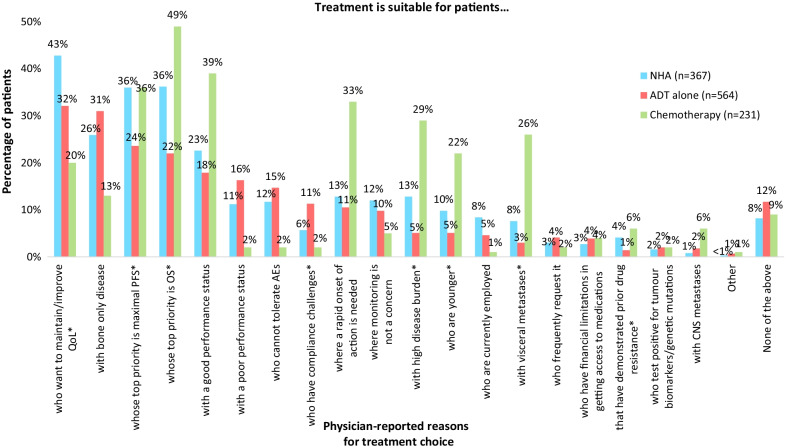
Physician-reported reasons for treatment choice: NHA or Chemotherapy [± ADT] vs ADT alone. *Note*: All treatments were ± ADT, except ADT alone. *Significant difference observed between treatment groups (*p* < 0.0167). *ADT* androgen deprivation therapy, *AE* adverse event, *CNS* central nervous system, *NHA* novel hormonal agents (abiraterone, enzalutamide, apalutamide, darolutamide), *OS* overall survival, *PFS* progression free survival, *QoL* Quality of Life

Pairwise analyses examining the ‘key clinical reasons for treatment choice’ (reasons with percentages ≥ 10% in the NHA [± ADT] group) revealed that physicians prescribed NHA (± ADT) vs ADT alone to significantly higher proportions of patients: who wanted to maintain/improve patients’ quality of life (QoL) (42.8%; n = 157 vs 32.1%; n = 181, *p* = 0.001); whose top priority was OS (36.2%; n = 133 vs 22.0%; n = 124, *p* < 0.0001) or maximal progression free survival (PFS) (36.0%; n = 132 vs 23.6%; n = 133, *p* < 0.0001); or who had high disease burden (12.8%; n = 47 vs 5.1%; n = 29, *p* < 0.0001). All ‘key clinical reasons for treatment choice’ for NHA (± ADT) vs ADT alone are presented in Fig. 2.

### *Key factors associated with chemotherapy use: chemotherapy (*± *ADT) vs ADT alone*

Patients receiving chemotherapy (± ADT) were significantly younger (67.4 ± 7.2 years) than patients receiving ADT alone (74.7 ± 7.7 years; *p* < 0.001) (Table 4). Examining the ‘key clinical reasons for treatment choice’ (reasons with proportions ≥ 10% in the chemotherapy [± ADT] group) revealed that physicians prescribed chemotherapy (± ADT) vs ADT alone to significantly higher proportions of patients who were younger: (22.1%; n = 51 vs 5.1%; n = 29, *p* < 0.0001), who had good performance status (39.4%; n = 91 vs 17.9%; n = 101, *p* < 0.0001), whose top priority was OS (49.4%; n = 114 vs 22.0%; n = 124, *p* < 0.0001), whose top priority was maximal PFS (35.9%; n = 83 vs 23.6%; n = 133, *p* < 0.0001), for whom a rapid onset of action was required (32.5%; n = 75 vs 10.5%; n = 59, *p* < 0.0001), who had high disease burden (29.4%; n = 68 vs 5.1%; n = 29, *p* < 0.0001), or who had visceral metastases (26.0%; n = 60 vs 3.0%; n = 17, *p* < 0.0001). All ‘key clinical reasons for treatment choice’ for chemotherapy (± ADT) vs ADT alone are presented in Fig. 2.

### *Key factors associated with ADT alone use: ADT alone vs chemotherapy (*± *ADT) or NHA (*± *ADT)*

Examining the ‘key clinical reasons for treatment choice’ (reasons with proportions ≥ 10% in the ADT alone group) revealed that physicians prescribed ADT alone vs chemotherapy (± ADT) to significantly higher proportions of patients with; a poor performance status (16.3%; n = 92 vs 2.2%; n = 5, *p* < 0.0001), who wanted to maintain/improve their QoL (32.1%; n = 181 vs 20.3%; n = 47, *p* = 0.001), who may have compliance challenges (11.3%; n = 64 vs 2.2%; n = 5, *p* < 0.0001), who could not tolerate adverse events (AEs) (14.7%; n = 83 vs 2.2%; n = 5, *p* < 0.0001), or with bone only disease (31.0%; n = 175 vs 12.6%; n = 29, *p* < 0.0001). The only significant difference between ADT alone vs NHA (± ADT) in terms of ‘key clinical reasons for treatment choice’ was for “treatment is suitable for patients who may have compliance challenges”, with physicians prescribing ADT alone rather than NHA (± ADT) to a significantly higher proportion of patients (11.3%; n = 64 vs 5.7%; n = 21, *p* = 0.004). All ‘key clinical reasons for treatment choice’ for ADT alone vs NHA or chemotherapy (± ADT) are presented in Fig. 2.

## Discussion

The main objective of the present real-world survey was to understand the impact of the approval of NHAs in the mHSPC setting in the US, EU5, and Japan. To the best of our knowledge, this was the first real-world survey evaluating NHA use in the mHSPC setting in different regions of the world. We found that globally, at the time of data collection, the most common mHSPC regimen initiated was still ADT alone (47%), followed by NHA (± ADT) (31%) and chemotherapy (± ADT) (19%). The low rate of chemotherapy use is unsurprising given the fact that docetaxel is only approved in the EU and at the time of data collection this approval was still relatively recent (i.e., September 2019). The highest rates of ADT alone usage were observed in Japan and Italy, and the lowest in Spain and in the US. Differences in ADT alone usage between these regions/countries should be considered in the context of integration into treatment guidelines and insurance coverage, although other factors (e.g., patient choice, physician awareness, preference and/or specialty, drug cost/reimbursement) may have also played a role.

Ng and colleagues [2] have recently provided a useful overview of the potential decision-making factors influencing choice of first line treatment for mHSPC (in the absence of head-to-head clinical trial data for NHAs in the mHSPC setting), including patient and disease factors, as well as drug licensing and reimbursement. Importantly, clinical trial populations diverge from patients in real-world practice, with trial populations including more de novo patients, whereas in the real-world many patients are likely to have received radical treatment prior to developing metachronous metastases. In addition, patients in the clinical setting tend to be older, less physically fit and with more comorbidities, and hence treatment decisions might be complicated by competing risks [2]. For this subset of patients who are older, less fit, and with more comorbidities, ADT alone remains a reasonable option, although treatment intensification with NHAs or chemotherapy now represents a new SOC in the management of mHSPC treatment in many developed countries, with docetaxel generally being reserved for patients with high-volume disease.

NHAs have been used in the mCRPC setting since the approval of abiraterone in 2011 [38]. These agents were then approved for men with mHSPC as early as November 2017 in Europe (abiraterone); although our international survey took place in 2020, some delay in change in practice patterns following these approvals and inclusion in guideline recommendations may naturally be expected. There might be an association between this delay and the results of this study. It is likely that NHA use in the mHSPC setting, and in the treatment of metastatic prostate cancer overall, will increase over time. Such trend was recently confirmed by Ke and colleagues [31]. Furthermore, our findings suggested that this might already be the case in the US, where the most common mHSPC regimen initiated was NHA (± ADT) (41%), closely followed by ADT alone (36%), and chemotherapy (± ADT) (12%).

Previous real-world investigations in the US, evaluating care in mHSPC patients who initiated treatment between 2014 and 2019, reported that overall (i.e., over the entire period), ADT alone was the most common mHSPC regimen initiated, with rates ranging from 47 to 63%. In contrast, NHA use over the same period was low (5–14%) [30–33], although this finding is unsurprising given that NHAs in the mHSPC setting did not receive US Food and Drug Administration approval until 2018–2019. Indeed, Ke et al. [31] reported that mHSPC patients in their 2017–2018 cohort were less often receiving ADT alone (43% vs 52%) and more often abiraterone (10% vs 4%) as initial regimen compared with the 2015–2016 cohort. Nevertheless, Swami et al. [32] recently pointed out that in 2018–2019, most men with mHSPC in the Optum health insurance claims database still received ADT alone, including those with visceral metastases (55%); the equivalent rate for NHA + ADT for the same group was 17%. Likewise, George et al. [4] reported that even in 2019, over half of mHSPC patients treated in real-world settings (oncology practices in the ConcertAI Oncology Dataset) did not initiate therapy now known to significantly improve survival (NHA + ADT or NHA + docetaxel) over ADT alone. Importantly, and in contrast with the present survey using patient data at a point-in-time in 2020, none of these previous investigations assessed the rate of NHA use in 2020, and instead they used broad data ranges from as early as 2014, which extended to prior to NHA approval for mHSPC in the US.

Although ADT alone still dominates the mHSPC treatment space outside the US, the use of NHAs is expected to increase in other countries/regions in the coming months and years. The speed of uptake of these new therapies in real-world settings, however, may be influenced by patient and disease factors, drug licensing, cost/reimbursement issues and physician awareness and education, as well as other local, regional, and national factors [2, 4, 39]. Fallara and colleagues, who evaluated three nationwide healthcare registries in Sweden, recently reported that uptake of the new indication for abiraterone in men with de novo mHSPC was low (12%) within 27 months after approval of the subsidized use of this agent, which indicates that even with subsidies uptake could be low in some countries or regions. The present survey found that NHA (± ADT) use differed across the EU5, with Spain, France, and Germany having the highest use, and Italy and the UK the lowest.

The second objective of this survey was to identify the key factors associated with NHA or chemotherapy (± ADT) usage compared with ADT alone, and we observed some similarities as well as differences in clinical decision making, based on factors such as patient fitness, compliance, patient preference, and tolerance of adverse events. Globally, physicians in our survey prescribed NHA or chemotherapy (± ADT) to younger patients who were able to tolerate more aggressive treatment, with the goal of extending life. Physicians reserved ADT alone for older patients who may have compliance issues and who were intolerant to AEs and, most importantly, whose goal was to maintain current QoL.

Likewise, Swami and colleagues [32] found that US patients in the Optum health insurance claims database who received chemotherapy (docetaxel) + ADT or NHA + ADT were younger (mean 68 and 73 years, respectively) than patients who received ADT alone (mean 75 years), although, unlike in the present survey, a greater proportion of patients with more aggressive disease (i.e., visceral metastases) received ADT alone (55%) vs chemotherapy + ADT (9%) or NHA + ADT (17%). Younger age in chemotherapy + ADT-treated men with mHSPC was also observed by Tagawa et al. [33], who also found that patients treated with NHA (abiraterone) + ADT or chemotherapy (docetaxel) + ADT were more likely to have metastatic disease in lymph nodes (also observed in our survey) and other sites at treatment initiation.

Fallara et al.’s [39] real-world investigation in Sweden indicated low adherence to the restriction that only men with high-risk mHSPC and men not suitable for docetaxel should receive abiraterone. By contrast, we found that, globally, NHA (± ADT) and chemotherapy (± ADT) rather than ADT alone were more commonly used in mHSPC with high-risk status, high disease volume, and distant metastases (largely in line with approved indications for abiraterone in the US, EU, and Japan [17, 22, 23], and apalutamide and enzalutamide in Japan [20, 24]), although we did not assess specific NHA use, as the approval timelines for specific NHAs vary, from late 2017 to 2020 in some countries/regions.

The mHSPC treatment landscape has changed considerably over the past few years and continues to evolve [8]. In future, increased NHA use in mHSPC patients may impact treatment patterns in the mCRPC setting, too. NHA rechallenge may become the frontline treatment option in mCRPC in countries where this is an acceptable option (e.g., Germany [40] and Japan [41]). However, in other countries, such as the UK [42] and France [43], it remains to be seen how the treatment patterns will evolve, since rechallenging NHA is not an approved treatment option and therefore chemotherapy (docetaxel) may be the first-line treatment of choice in the mCRPC setting. Future studies are warranted to assess how the treatment patterns will evolve within the metastatic prostate cancer setting.

Several limitations should be considered in the interpretation of our findings. The DSP was not based on a true random sample of physicians or patients. Although the selection of participating physicians was based on minimal inclusion criteria, participation was influenced by their willingness to complete the survey. Physicians were asked to provide data for a consecutive series of patients to avoid selection bias, but no formal patient selection verification procedures were in place. In addition, we assessed perceived key clinical reasons for treatment choice, but other reasons may exist, including perceived key reasons not being applicable uniformly to all physicians, or patients participating in the survey not reflecting the general mHSPC population.

Another limitation of this analysis was that the Bonferroni correction was not applied to the results, which could have led to issues surrounding the family-wise type 1 error. Therefore, care should be taken when interpreting the results when looking for significance. Furthermore, recall bias, a common limitation of surveys, may have also affected physicians’ responses to the questionnaires. On the other hand, data for these analyses were collected at the time of each patient’s appointment and this was expected to reduce the likelihood of recall bias.

Finally, it is important to acknowledge that only developed countries were surveyed and therefore the findings of this study may not be generalisable to developing countries, which may face different challenges in the treatment of prostate cancer patients.

Despite these limitations, real-world studies play an important role in identifying areas of concern that are not usually addressed in randomised controlled trials (RCTs). Compared with RCT populations, real-world studies include more heterogenous samples, which are more reflective of real-world clinical practice. As such, real-world data can complement clinical trial evidence and provide insight into the effectiveness of interventions in patients commonly seen in clinical practice.

## Conclusions

Until five years ago, the mHSPC treatment space was dominated by the use of ADT alone. However, novel agents have been approved since late 2017. The present survey found that, globally, at the time of data collection (January–August 2020), ADT alone was still the most common mHSPC treatment regimen initiated first, suggesting that physicians may prefer using treatments that they are familiar with and have experience with (although there are many other factors that may impact prescribing practice), despite clinical trial evidence of improved survival with NHA or chemotherapy (± ADT) vs ADT alone. This survey also indicated that physicians prescribed different mHSPC treatments based on specific criteria, including patient preference, disease burden/severity, and the fitness of the patient. Lastly, our survey offered an early look at the evolving mHSPC treatment paradigm, and it may be that physicians need to better understand the benefit:risk ratios of NHA (± ADT) over ADT alone before they begin to utilize these newer therapies routinely. In order to fully appreciate the rapidly changing mHSPC treatment landscape and monitor NHA uptake specifically, additional real-world studies are required. Future research could also evaluate the impact of NHA use in the mHSPC setting on treatment patterns in the mCRPC patients.

## Data Availability

The data reported in this study were derived from an independent survey (the Adelphi Real World Prostate Cancer IV DSP). All data, i.e. methodology, materials, data and data analysis, supporting the study are the intellectual property of Adelphi Real World. The data that support the findings of this study are available from Adelphi Real World but restrictions apply to the availability of these data, which were used under license for the current study, and so are not publicly available. Data are however available from the authors upon reasonable request and with permission of Adelphi Real World. All requests for access should be addressed directly to Andrea Leith at andrea.leith@adelphigroup.com.

## Abbreviations

ADT: Androgen deprivation therapy
AE: Adverse event
Chemo: Chemotherapy
DSP: Disease specific programme
ECOG: Eastern cooperative oncology group
EU: European Union
EU5: France, Germany, Italy, Spain, and the United Kingdom
mCRPC: Metastatic castration-resistant prostate cancer
mHSPC: Metastatic hormone-sensitive prostate cancer
NCCN: National comprehensive cancer network
NHA: Novel hormonal agents
OS: Overall survival
PFS: Progression free survival
PRF: Patient record form
PSA: Prostate-specific antigen
QoL: Quality of life
SD: Standard deviation
SOC: Standard of care
UK: United Kingdom
US: United States
